# Auraptene inhibits migration, invasion and metastatic behavior of human malignant glioblastoma cells: An *in vitro* and *in silico* study

**DOI:** 10.22038/AJP.2023.23586

**Published:** 2024

**Authors:** Seyed Hadi Mousavi, Mohammad Jalili-Nik, Mohammad Soukhtanloo, Arash Soltani, Farzaneh Abbasinezhad-Moud, Hamid Mollazadeh, Farzaneh Shakeri, Bahram Bibak, Amirhossein Sahebkar, Amir R. Afshari

**Affiliations:** 1 *Medical Toxicology Research Center, Mashhad University of Medical Sciences, Mashhad, Iran*; 2 *Department of Clinical Biochemistry, Faculty of Medicine, Mashhad University of Medical Sciences, Mashhad, Iran*; 3 *Student Research Committee, Mashhad University of Medical Sciences, Mashhad, Iran*; 4 *Natural Products and Medicinal Plants Research Center, North Khorasan University of Medical * *‎* *Sciences, Bojnurd, Iran*; 5 *Center for Global Health Research, Saveetha Medical College and Hospitals, Saveetha Institute of Medical and Technical Sciences, Saveetha University, Chennai, India *; 6 *Applied Biomedical Research Center, Mashhad University of Medical Sciences, Mashhad, Iran *; 7 *Biotechnology Research Center, Pharmaceutical Technology Institute, Mashhad University of Medical Sciences, Mashhad, Iran*

**Keywords:** Auraptene, Glioblastoma multiforme, Migration, Invasion, Metastasis

## Abstract

**Objective:**

The present work examined the anti-metastatic effects of auraptene and their underlying mechanisms of action in U87 Glioblastoma multiforme (GBM) cells.

**Materials and Methods:**

To test the hypothesis, cell culture, Matrigel invasion assay, scratch wound healing assay, gelatin zymography assay, qRT-PCR, and western blot experiments were conducted.

**Results:**

At sublethal concentrations of 12.5 and 25 µg/ml, auraptene exhibited a significant reduction in cell invasion and migration of U87 cells, as assessed using scratch wound healing and Transwell tests, respectively. The qRT-PCR and zymography experiments demonstrated a significant decrease in both mRNA expression and activities of MMP-2 and MMP-9 following auraptene treatment. Western blot analysis also showed that MMP-2 protein level and phosphorylation of metastasis-related proteins (p-JNK and p-mTOR) decreased in auraptene-treated cells. Molecular docking studies consistently demonstrated that auraptene exhibits a significant affinity towards MMP-2/-9, the ATP binding site of mTOR and JNK1/2/3.

**Conclusion:**

Auraptene inhibited the migration and invasion of GBM cells. This inhibitory effect was induced by modulating specific mechanisms, including suppressing MMPs, JNK, and mTOR activities.

## Introduction

**Figure 1:**
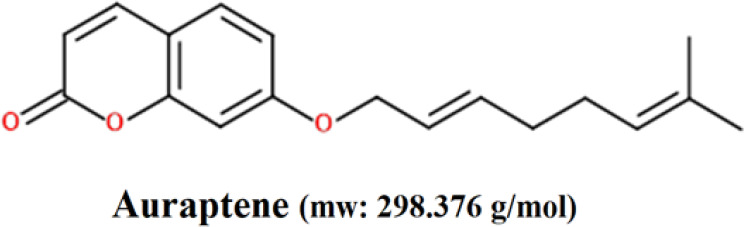
Chemical structures of auraptene

Auraptene (7-geranyloxycoumarin, depicted in Figure 1) is categorized as a member of the Rutaceae botanical family and is known for having the highest concentration of prenyloxycoumarin compounds observed in nature, based on the available data (Sahebkar, 2016). Some recent discoveries have shown that auraptene may be a potent antitumor agent (Derosa et al., 2016; Tanaka et al., 2010b). Anti-invasive and anti-metastatic impacts of auraptene have been exhibited in some human cell lines, including cervical, ovarian, and breast tumors (Kawabata et al., 2006b; Tanaka et al., 2010a; Tanaka et al., 1998). 

Glioblastoma multiforme (GBM) constitutes more than 50 percent of glioma incidences and is the most diverse, severe, and lethal primary neoplasm of the brain in the adult population (Maghrouni et al., 2021; Sahab-Negah et al., 2020). The standard treatment for GBM often involves a combination of safe maximal surgical resection, chemotherapy with the alkylating drug temozolomide, and radiation (Jalili-Nik et al., 2020). However, the prognosis for GBM remains unfavorable due to its aggressive nature characterized by fast growth, extensive infiltration, local spread, and resistance to treatment (Mollazadeh et al., 2020; Sahab-Negah et al., 2020). Therefore, finding a new, less toxic, and more effective GBM therapeutic agent seems necessary (Afshari et al., 2021a; Afshari et al., 2021b; Afshari et al., 2021c; Ahamed et al., 2023; Foroutan et al., 2022; Sanati et al., 2022). In our earlier study, we provided evidence that auraptene elicits cytotoxic effects by promoting the formation of reactive oxygen species. Additionally, we observed that auraptene promotes apoptosis by upregulating the Bax/Bcl-2 ratio (Afshari et al., 2019a; Afshari et al., 2019c). However, the exact impacts and possible molecular mechanisms of auraptene on the metastasis of U87 cells remain unknown.

Critical parts of the metastatic process in GBM include proliferation, angiogenesis, invasion, and migration (Xie et al., 2014). The process of metastasis necessitates the breakdown of components within the extracellular matrix (ECM) via the action of proteolytic enzymes, specifically metalloproteinases (MMPs) such as MMP-2 and MMP-9. The expression levels of MMPs, which are enzymes abundantly present in various tumor types, exhibit a favorable correlation with the invasion, migration, and metastasis of GBM (Munaut et al., 2003). Growing reports have shown that auraptene could suppress the invasion/migration of various cervical, breast, and ovarian cancers by downregulating MMPs (Charmforoshan et al., 2019b; Jamialahmadi et al., 2018). In addition to MMPs, c-Jun N-terminal kinase/stress-activated protein kinase (JNK/SAPK) and mammalian target of rapamycin (mTOR) are related to elevated tumor growth, proliferation, survival, invasion, and metastasis (Okamura et al., 2015; Prasad et al., 2011). The evidence has shown that inhibition of mTOR signaling induces the expression of nuclear factor-kappa B (NF-κB)-mediated proinflammatory cytokines, such as interleukin (IL)- 23, IL-12, IL-6, and tumor necrosis factor-α (TNF-α) in myeloid dendritic cells (Ohtani et al., 2008a; Ohtani et al., 2008b; Schmitz et al., 2008; Soltani et al., 2018; Weichhart et al., 2008; Yang et al., 2006). 

This study aims to provide novel perspectives on the effects of auraptene on the invasion, migration, and metastasis of GBM. Moreover, our study sought to investigate the underlying signaling pathways through which auraptene exerts its anti-metastatic effects on the human malignant GBM cell line. The research findings presented in this study enhance our understanding of the underlying mechanisms involved and provide a robust basis for the possible clinical utilization of auraptene in managing GBM.

## Materials and Methods

**Cell culture**

The U87 cell line originated from a human malignant GBM and was obtained from the National Cell Bank of Iran. The cells were cultured in Dulbecco's Modified Eagle Medium (DMEM) supplemented with high glucose, 100 U/ml of penicillin, 100 mg/ml of streptomycin, and 10% fetal bovine serum (FBS). The experimental protocol entailed maintaining the culture conditions at a temperature of 37°C within a humidified environment containing 5% CO_2_, following the guidelines outlined by the National Cell Bank of Iran.

**Matrigel invasion assay**

ECMatrix Collagen-based cell invasion assay was used to evaluate the anti-invasive ability of auraptene (Ling et al., 2016). The test was done in invasion plates based on the Boyden Chamber principle (Thomsen and Lade Nielsen, 2011). U87 cells were incubated overnight in an FBS-free medium containing auraptene at 12.5 and 25 μg/ml concentrations. The bottom compartment of the chamber was filled with DMEM containing 10% FBS, which served as a chemoattractant. U87 cells invaded and crossed the polymerized collagen layer and clung to the bottom of the polycarbonate membrane. 

After that, the wells were treated with CyQuant GR® dye and allowed to stain for 20 min at room temperature. Subsequently, the invading cells were observed and quantified using a light microscope (Zeiss Axiovert® 200) at a magnification of 5x. Each treatment was carried out in triplicate, and a total of three separate experiments were conducted.

**Scratch wound healing assay**

An *in vitro* scratch wound healing assay was employed to assess the anti-migratory potential of auraptene.(Liang et al., 2007). In brief, 7×10^5^ cells were grown in 6 well plates, and a monolayer of cells was scratched with a 100-μl pipette tip to create a wound. Next, floating cells were removed after washing with phosphate buffer saline (PBS), and the FBS-free medium containing auraptene (12.5 and 25 μg/ml) was added to the wells. After 4, 24, and 48 hr, the migration of cells was photographed at 5x magnification using a light microscope Zeiss Axiovert^®^ 200. Each value was derived from three randomly selected fields, and outcomes are indicated as the gap closure distance (μm) per field. All treatments were conducted in triplicate, and three independent experiments were done.

**Gelatin zymography assay**

The gelatinolytic activities of MMP-2 and MMP-9 ‎were investigated using gelatin zymography (Sato et al., 1994). In brief, the U87 cells were treated with the IC_50_ concentration of auraptene (100 μg/ml, according to our previous study) (Afshari et al., 2019e) for 24 and 48 hr. The supernatant from the culture was centrifugated, and 30 µg of the resulting supernatant was electrophoresed on a 10% polyacrylamide gel containing gelatin (1 mg/ml) as a substrate. The gel was washed every 20 min using a solution containing Triton X-100 to eliminate sodium dodecyl sulfate (SDS). Subsequently, it was incubated for 36 hr at 37℃ in an appropriate incubation buffer (consisting of 1 μM ZnCl_2_, 50 mM Tris-HCl [pH 7.5], 200 mM NaCl, and 5 mM CaCl_2_), and the gel was stained with Coomassie Brilliant Blue R-250 for 30 min. The image of the gels was obtained using a GS-800 calibrated densitometer (Bio-Rad, HC, USA). The analysis was performed using Image J 1.52a software (NIH, Bethesda, Rockville, MD, USA), and the results were compared with the control groups.

**qRT-PCR**

qRT-PCR was used to evaluate the mRNA expression of *MMP-2* and *MMP-9*, as reported in our previous studies (Afshari et al., 2019d). U87 cells were seeded and subsequently exposed to auraptene at 100 µg/ml concentration for 24 hr. Then, total RNA was extracted according to the RNeasy^®^ mini kit protocol (Qiagen GmbH, Hilden, Germany) (Dawoody Nejad et al., 2017). Total RNA was reverse-transcribed (TaKaRa Holdings, Inc., Kyoto, Japan), and qRT-PCR was performed using specific primers for *MMP-2*, *MMP-9*, and *GAPDH* (as housekeeping gene) (Macrogene Co., Seoul, South Korea, Table 1), Real-time PCR Master Mix without Rox™ (Amplicon, Denmark), and a Light-Cycler 96 Real-time PCR for the amplification (Roche, USA). The Livak method (2^−ΔΔCt^) was utilized to analyze the relative expression of target genes.

**Table 1:** The sequence of primers in the present study

**Gene symbol**	**Gene name **	**Primer sequence (5ʹ → 3ʹ)**
***GAPDH***	*Glyceraldehyde-3-phosphate dehydrogenase*	*Forward: TCAAGATCATCAGCAATGCCTCC* *Reverse: GCCATCACGCCACAGTTTC *
***MMP-2***	*Matrix metalloproteinase 2*	*Forward: TGGCAAGTACGGCTTCTGTC * *Reverse:* *AGCTGTCATAGGATGTGCCC *
***MMP-9***	*Matrix metalloproteinase 9*	*Forward: GCATAAGGACGACGTGAATGG * *Reverse: TGTGGTGGTGGTTGGAGG *

**Preparation of lysates, protein extraction, and western blot analysis**

Western blotting was used to evaluate the protein expression of essential metastasis-related genes (Afshari et al., 2019a). In this part, 7 × 10^5 ^U87 cells were treated with 50 and 100 µg/mL of auraptene for 24 hr to assess MMP-2, MMP-9, and NF-κB p65 protein levels. U87 cells were also seeded, and the protein expression of JNKs, phospho-JNK, mTOR, and phospho-mTOR was determined after 0, 15, and 60 min of treatment with 100 µg/mL of auraptene. For the next step, cells were lysed in radioimmunoprecipitation assay (RIPA) lysis buffer (250 mM Tris-HCl, pH 7.4, 750 mM NaCl, 5 mM ethylenediaminetetraacetic acid (EDTA), 5% Triton X-100, 0.5% SDS) and kept on ice for 30 min. The cellular lysates were subjected to separation using 8-12% SDS-PAGE, followed by their transfer onto a polyvinylidene difluoride (PVDF) membrane using a glycine transfer buffer. The membrane was then blocked and incubated with primary antibodies, followed by a secondary antibody in Tris-buffered saline (TBS) containing 0.1% Tween-20 and bovine serum albumin (BSA). The Super Signal® West Femto western blotting kit (Thermo Fisher Scientific, Inc., USA) was employed to detect each protein, following the guidelines provided by the manufacturer. The protein expression levels were assessed using Image J 1.52a software (NIH, Bethesda, Rockville, MD, USA). The obtained data were then compared to the expression of beta-actin protein. 

**Molecular docking**

For the molecular docking studies, the six co-crystal structures of target proteins JNK1 ‎‎(protein data bank [PDB] ID: 3ELJ), JNK2 (PDB ID: 3E7O), JNK3 (PDB ID: 6EMH), MMP-2 (PDB ID: ‎‎1HOV), MMP-9 (PDB ID: 4XCT), and mTOR (PDB ID: 4JT6) in complex with their respective inhibitors were obtained ‎from the Protein Data Bank (https://www.rcsb.org) (Yau et al., 2019). Docking was performed using Genetic ‎Optimization for Ligand Docking (GOLD) Suite 5.2.2 software (the Cambridge ‎Crystallographic Data Centre [CCDC], Cambridge, UK). 

Before the docking procedure, the crystal structures were prepared by adding hydrogen atoms, rectifying residues with absent atoms and side chains, and removing water molecules and ligands. In order to assess the reliability of the docking operation, the GOLD program underwent testing by docking the co-crystallized ligands back into the binding pocket of their respective structures. The default settings for the genetic algorithm parameters were maintained, except for the population size and number of operations, which were increased to 1000 and 1,000,000, respectively. The scoring was based on the ChemScore fitness, and the highest-scoring pose of each ligand was extracted for further analysis using the Discovery Studio Visualizer 4 (Accelrys, CA, USA).

**Statistical analysis**

The results underwent triple analysis compared to control cells that were not treated, utilizing GraphPad Prism® ‎‎7.01 software (GraphPad Software, San Diego, CA, USA). The collected data was assessed using A one-way analysis of variance (ANOVA) followed by the Dunnett test to compare the values. Values below 0.05 were deemed statistically significant. The results are presented as the mean±standard deviation.

## Results

**Auraptene exhibits a significant inhibitory effect on the invasion and migratory capabilities of U87 cells**

**Figure 2:**
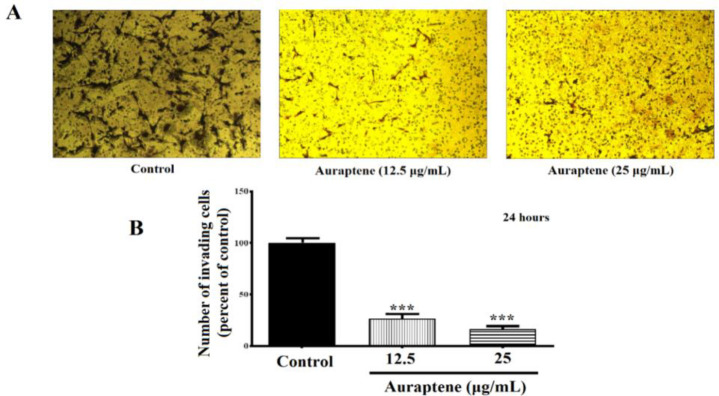
The number of invading cells treated by auraptene using an invasion assay. Auraptene was evaluated at concentrations below IC_50_ (12.5 and 25 μg/ml) for 24 hr. (A) represents cellular invasion at two concentrations under the reversed Zeiss Axiovert 200 microscope with a magnification of 5x. (B) Demonstrates the cell invasion that decreased significantly in both concentrations at 24 hr. Statistical analysis was performed using the software GraphPad Prism^®^ 7.01, and results were compared with those of the control group. Each column represents the mean ± standard error in the sample. p<0.001*** compared to the control group (n=3).

The principal aspect of the high recurrence rates of GBM is its high invasive potential (Connor et al., 2007); therefore, with a Matrigel^®^ invasion experiment, the effects of auraptene on U87 cell invasion were tested. Figure 2 displays illustrative photographs depicting the invasion of U87 cells. The number of invading cells exhibited a considerable decrease, reaching 26.5 and 16.35% following treatment with auraptene at concentrations of 12.5 and 25 µg/ml for a duration of 24 hr, respectively (p = 0.0007 at concentration of 12.5 µg/ml, and p=0003 at concentration of 25 µg/ml). These results showed that auraptene mitigated the motility of U87 cells considerably in a concentration-dependent manner. 

**Figure 3:**
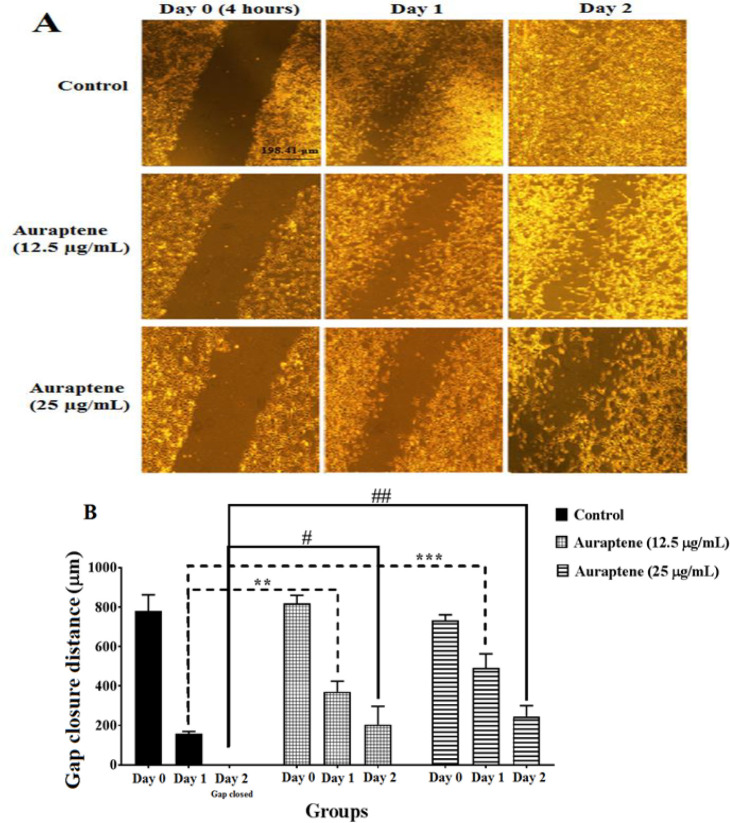
U87 cell migration was assessed by wound-healing assay at different times. Auraptene was evaluated at concentrations below IC_50_ (12.5 and 25 μg/ml) at 4 (day 0), 24 (first day), and 48 (second day) hr after treatment. (A) represents cell migration regions at different times by reverse microscope Zeiss Axiovert^®^ 200 with a magnification of 5x. (B) Statistical analysis was performed using GraphPad Prism® 7.01, and results were compared with those of the control group. It was observed that the amount of cell migration in the first and second days after auraptene treatment was significantly decreased in both concentrations. Each column represents the mean ± standard error in the sample. p<0.01** and p<0.001***as well as p<0.05^#^, and p<0.01^##^ as compared to the control group (n=3).

The leading cause of death in most cancer patients is metastases in rapidly migrating tumor cells; thus, a practical approach could prevent tumor metastasis by inhibiting cell migration. To investigate this probability further, we conducted a secondary analysis by subjecting the cells to auraptene treatment at 12.5 and 25 µg/ml doses for 24 and 48 hr. Subsequently, a wound-healing assay was performed to evaluate the impact of auraptene on cell migration. As shown in Figure 3A, the migration of cells was diminished after exposure to auraptene in a concentration-dependent manner following 24 hr of treatment. An identical occurrence was observed after a 48-hr treatment period (Figure 3B, day 1: 0.0031 at concentrations of 12.5 μg/ml, and p=0.0009 at concentrations of 25 μg/ml, day 2: p=0.0112 at concentrations of 12.5 μg/ml, and p=0.007 at concentrations of 25 μg/ml, as compared to the control belonging to the same day). The outcomes demonstrated that auraptene strongly suppresses the invasion and migration of U87 cells.

**Effect of auraptene treatment on metastasis-related biomarkers of U87 cells**

**Figure 4:**
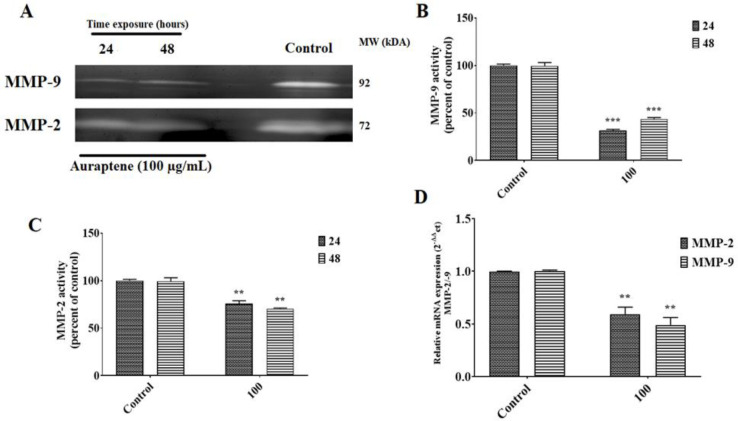
. (A, B, and C) Effect of auraptene on the MMP-2/MMP-9 activity of U87 cells. Cells were treated with IC_50_ concentration (100 µg/ml) of auraptene for 24 and 48 hr (A). The MMP-2 and MMP-9 activities were examined by gelatin zymography assay. MMP-2 and MMP-9 activities were quantified by densitometric analysis. The densitometric data are expressed as mean ± standard error of three independent tests (B and C). p<0.001 *** and p<0.01 compared to the control group. (D) The U87 cells were treated with a 100 μg/mL concentration of auraptene for 24 hr. Total RNA was extracted, and qRT-PCR analyzed mRNA expression. The relative gene expression levels of *MMP-2* and *MMP-9* were determined by 2^−ΔΔCt^ method analysis. The y-axis indicates the fold change. Results were normalized to levels of *GAPDH* in the samples. p<0.01** as compared to the control group.

**Figure 5:**
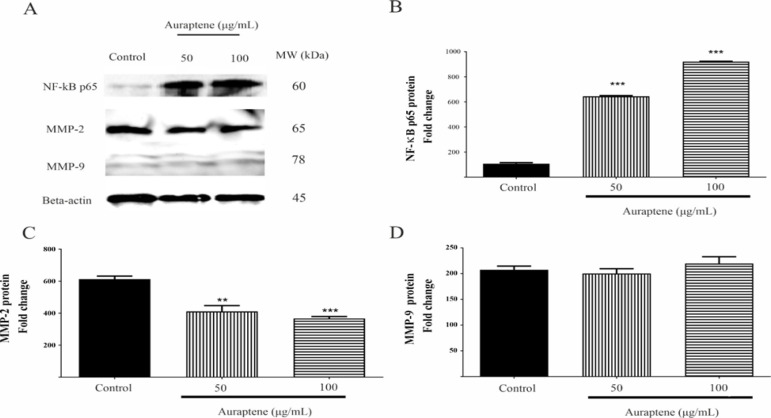
Auraptene induces NF-κB p65 protein expression and inhibits MMP-2 protein expression in U87 cells ‎‎(A). U87 cells were treated with 50 and 100 μg/mL of auraptene for 24 hr. The total protein levels of NF-κB ‎p65, MMP-2, and MMP-9 were assessed by western blotting and calculated. The relative density of NF-κB p65 ‎‎(B), MMP-2 (C), and MMP-9 (D) were determined by densitometry of the blots using Image J 1.52a software and ‎were compared to the beta-actin protein. **p<0.01 and ***p<0.001 compared to the control group.‎

In order to get a deeper comprehension of the mechanism by which auraptene inhibits cell invasion and migration in GBM cells, we analyzed the expression and activity levels of MMP-2 and MMP-9. First, a gelatin zymography analysis was performed to evaluate the enzymatic activities of MMP-2 and MMP-9. Figure 4A-C demonstrates a notable reduction in the activity of MMP-2 and MMP-9 in the serum-free medium following treatment with 100 μg/ml of auraptene for 24 and 48 hr. Second, we wanted to determine if a reduction in mRNA and protein levels caused a decrease in the enzymatic activities of MMP-2 and MMP-9. To investigate this, we exposed the cells to 50 and 100 μg/ml of auraptene for 24 hr and conducted western blotting and qRT-PCR analyses on the *MMP-2* and *MMP-9* genes. In comparison with the control treatment, the administration of auraptene at a concentration of 100 μg/ml resulted in a significant decrease in the expression of *MMP-2* and *MMP-9* genes at the mRNA level (Figure 4D, p=0.0094 for *MMP-2*, and p=0.002 for *MMP-9*). In addition, it was shown that the relative expression level of MMP-2 protein was substantially reduced in U87 protein lysates as compared to the untreated group (Figure 5C, p=0.0012 at a concentration of 50 μg/ml, and p=0.0007 at a concentration of 50 μg/ml). However, the MMP-9 protein expression was unchanged in auraptene-treated cells (Figure 5D).

**Auraptene upregulates NF-κB p65 protein and decreases the phosphorylation of JNK and mTOR proteins in U87 cells**

We analyzed the phosphorylation of JNKs, mTOR, and the expression of NF-κB p65 proteins to better understand the mechanism by which auraptene inhibits metastasis. ‎

**Figure 6:**
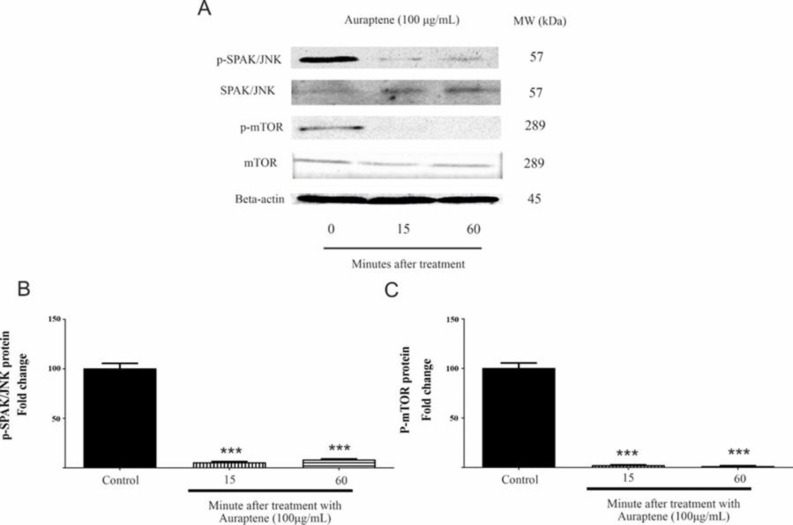
Auraptene decreases the phosphorylation of mTOR and JNK proteins in U87 cells (A). U87 cells ‎were treated with 100 μg/ml of auraptene for 15- and 60-min. Western blotting assesses and calculates the protein levels of mTOR, p-mTOR, JNK ‎‎, and p-JNKs. The relative density of p-JNKs (B) and p-mTOR (C) is ‎determined by densitometry of the blots using Image J 1.52a software and was compared to the beta-actin ‎protein as a loading control. Outcomes from three repeated and separate tests were similar. ***p<0.001 as ‎compared to the control group.‎

After 24-hr treatment of U87 cells with auraptene (50 and 100 μg/ml), a remarkable elevation was observed for NF-κB p65 protein expression (Figure 5B, p<0.0001). Furthermore, the U87 cell line was subjected to treatment with a concentration of 100 μg/ml of auraptene for a duration ranging from 0 to 60 min. Subsequently, western blot studies were conducted to assess the phosphorylation status of proteins. As shown in Figure 6B, auraptene significantly decreased the phosphorylation of JNKs after 15 and 60 min (p<0.0001). Also, auraptene inhibited the protein phosphorylation of mTOR (Figure 6C, p<0.0001). 

**Molecular docking analysis**

**Figure 7:**
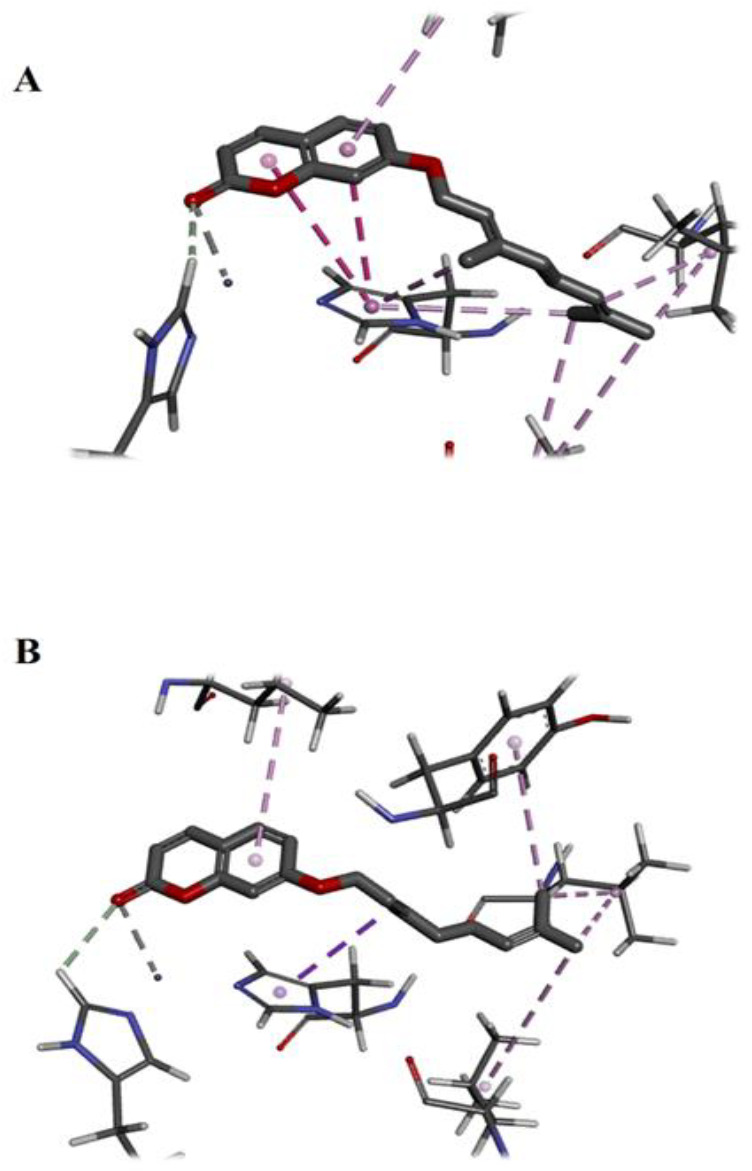
The binding mode of auraptene in MMP-2 (A) and MMP-9 (B). In both poses, auraptene interacted with zinc ions through the oxygen of chromen-2-one moiety.

To investigate the binding affinity, we docked auraptene into crystal structures of mTOR, MMP-2/-9, and JNK1/2/3. Table 2 displays the projected binding free energies (Chemscore.dG) and ChemScore functions (ChemScore.fitness) of the best position of docked auraptene and the re-docked co-crystallized inhibitors into the binding site of mTOR, JNKs, and MMPs. ‎Chemscore.dG values were transformed into predicted K_i_ values using the following formula: ΔG_binding_ = RTlnK_i_ (T, 300 K) for selectivity comparison. The results showed that the binding affinity of auraptene to mTOR, MMP-2, and MMP-9 was high, but it was less than the corresponding co-crystallized inhibitors. The binding mode of auraptene in MMP-2 and MMP-9 revealed that auraptene interacted with zinc ions through the oxygen of chromen-2-one moiety (Figure 7). 

Auraptene had a greater affinity for binding to JNK1 and JNK2 than the inhibitors co-crystallized with them. The results of the docking analysis revealed that the interaction between auraptene and JNK3 demonstrated a significantly greater affinity (predicted Ki ratio, 43.84) compared to the interactions between JNK1 and JNK2 (9.75 and 1.48, respectively). 

**Figure 8:**
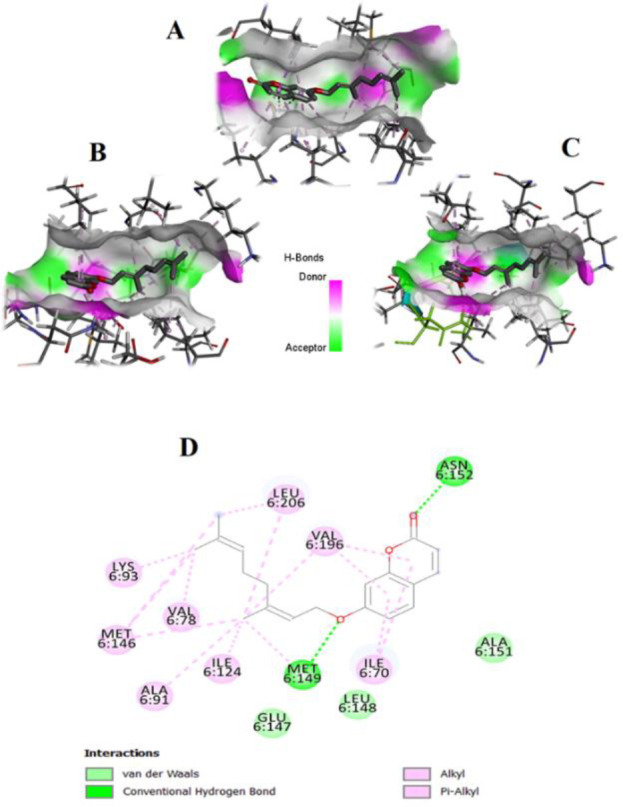
The binding mode of auraptene in JNK1 (A), JNK2 (B), and JNK3 (C).

This suggests that auraptene possesses a notably higher binding affinity for JNK3 than its co-crystallized inhibitor. Figure 8 displays the interaction mechanism of auraptene with JNK1 (A), JNK2 (B), and JNK3 (C), as well as the receptor surface of the hydrogen bond acquired from the docking outcomes.

**Table 2:** Predicted binding affinities of docked auraptene into the active site of JNKs, MMPs, and mTOR structures. ‎Auraptene was docked into the active site of the targets by GOLD docking software with the Chemscore fitness ‎function. As predicted binding free energies, DG values were converted to predicted Ki values ‎according to the following formula: ΔGbinding = RTlnKi (T, 300 K) for selectivity comparison. ‎Original, as defined by co-crystallized inhibitor in complex with the corresponding PDB structure.‎

Target proteins	PDB ID	Inhibitor	ChemScore.fitness	ChemScore.DG (kJ/mol.)	Predicted *K*_i_ Ratio (auraptene/original)
JNK1	3ELJ	Original	27.48	-30.51	9.75
		Auraptene	34.73	-36.19	
JNK2	3E7O	Original	37.43	-38.42	1.48
		Auraptene	38.26	-39.39	
JNK3	6EMH	Original	28.49	-29.51	43.84
		Auraptene	36.10	-38.94	
MMP-2	1HOV	Original	45.26	-51.83	0.01
		Auraptene	36.53	-40.25	
MMP-9	4XCT	Original	37.83	-49.63	0.08
		Auraptene	41.28	-43.43	
mTOR	4JT6	Original	38.96	-40.36	0.33
		Auraptene	34.20	-37.66	

**Figure 9:**
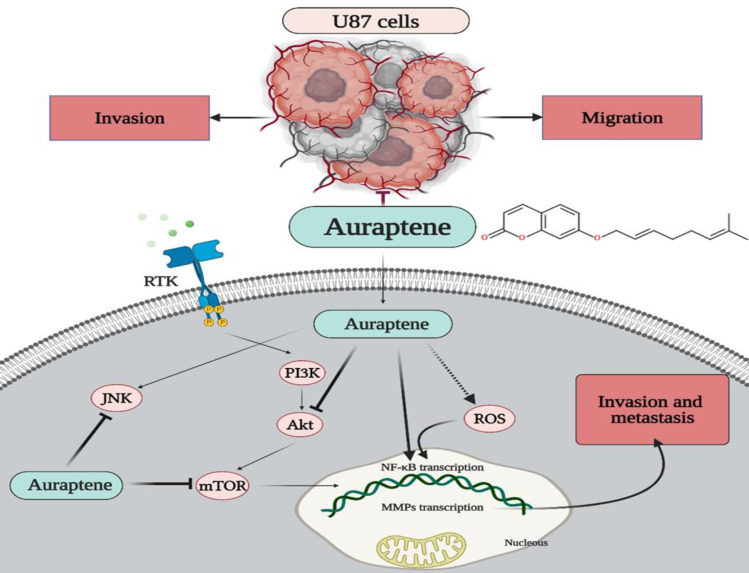
The proposed signaling pathways via which auraptene inhibited migration, invasion, and metastatic behavior of human malignant U87 cells. Collectively, auraptene diminished protein phosphorylation and subsequent inactivation of JNK and mTOR, contributing to reduced expression and activity of MMPs.

## Discussion

Glioblastoma multiforme (GBM), an exceedingly malignant neoplasm of the brain, presents a substantial public health issue owing to its elevated fatality rates, which persist unaltered despite the administration of rigorous chemotherapy and radiation therapies (Song et al., 2018; Tang et al., 2017). Temozolomide and bevacizumab (a vascular endothelial growth factor inhibitor) have been widely regarded as the primary therapeutic choices for managing GBM and mitigating its potential relapse (Afshari et al., 2020). Regrettably, the efficacy of these medications in avoiding local metastasis of GBM is significantly limited, as indicated by low response rates (Chinot et al., 2014; Hovinga et al., 2019). Considering the limited effectiveness of existing treatments, it is crucial to explore new pharmaceutical approaches to improve the prognosis of patients (Ghorbani et al., 2023; Hosseini et al., 2023; Izadi et al., 2023; Roshan et al., 2023). Hence, the primary objective of this study was to investigate the potential anti-metastatic properties of auraptene, marking the first instance of such an examination. According to our previous study, auraptene IC_50_ was determined at about 108.9 μg/mL for 24 hr of treatment in U87 cells (Afshari et al., 2019c). 

We found that auraptene mechanistically inhibits migration, invasion, and metastasis, as revealed by decreasing MMPs activity and modulation of mTOR/JNK and NF-κB pathways. The findings from our earlier work demonstrated that auraptene exhibits potent cytotoxic effects and induces cellular death (Afshari et al., 2019b; Afshari et al., 2019c). Also, studies have confirmed that auraptene inhibits metastasis in specific tumor cells (Charmforoshan et al., 2019a; Kawabata et al., 2006a; Moon et al., 2015); however, whether auraptene suppressed metastasis in GBM cells has yet to be elucidated. 

ECM degradation invades and disseminates human GBM into the normal brain parenchyma, accomplished by MMPs secretion from cancerous cells (Lim et al., 2020). MMPs, as essential contributors to cancer cell invasion and dissemination, are considered critical players in tumor metastasis, migration, and invasion, and agents that inhibit the proteolytic activity of MMPs can suppress cancer cell metastasis (Castro-Castro et al., 2016). Also, previous research has reported on the involvement of proteases MMP-2 and MMP-9 in the processes of invasion, migration, and metastasis in developing GBM (Roshan et al., 2019b). Hence, metastasis, cell migration, and invasion of surrounding tissues are among the essential attributes of GBM (Jiang et al., 2019). The proteins MMP-2 and MMP-9 have crucial functions in facilitating the process of metastasis. Both genes demonstrate substantial expression at both the messenger RNA and protein levels during the development of GBM (Rao, 2003), which are also acknowledged as prognostic indicators for an unfavorable prognosis of GBM (Nakada et al., 2003). Hopefully, the results of the current manuscript revealed that auraptene could diminish the metastatic behavior of U87 cells by suppressing the MMP-2 protein expression, *MMP-2/-9* mRNA expression, and enzymatic activity of MMP-2/-9, suggesting that auraptene might be a beneficial agent against invasion and metastasis of GBM. In agreement with our experimental results, the docking data provided essential information ‎about the positioning and binding orientations of the auraptene into the active site of MMP-‎‎2/-9. 

JNKs and mTOR are the essential regulators of physiological and pathological processes, ‎which are hyper-activated in multiple cancers (Paquette et al., 2018; Roshan et al., 2019a), particularly GBM (Huang et al., 2018). Several ‎studies have indicated that mTOR complex and JNKs are upstream regulators of MMP-2 and ‎MMP-9, crucial in cell invasion and dissemination (Chen et al., 2014). The study conducted by Cheng et al. showed that the inhibition of ERK and JNK-mediated MMPs production effectively suppresses metastasis (Cheng et al., 2016; Lee et al., 2015). To investigate the impact of auraptene on the upstream regulators of MMPs, we evaluated the phosphorylation levels of JNK and mTOR proteins in GBM cells. Auraptene demonstrated a notable capacity to decrease the levels of protein phosphorylation in JNK and mTOR. Besides, our *in-silico* results indicated that auraptene efficiently docked in the active ‎site of mTOR and JNKs with high ChemScore functions compared with their respective inhibitors as a ‎gold standard. Based on the predicted Ki ratio (auraptene/co-crystallized inhibitor), the binding ‎affinity of auraptene to the active site of JNK3 was higher than JNK1/2. So, it can be concluded ‎that auraptene might be an anti-metastatic and anti-invasiveness natural agent against U87 ‎cells, possibly through regulating phosphorylation of JNK and mTOR proteins and suppressing MMP-2 and ‎MMP-9 expression and activity. 

‎ Numerous studies have documented that the suppression of mTOR signaling leads to the upregulation of proinflammatory cytokines mediated by NF-κB, such as TNF-α, IL-12, IL-23, and IL-6, in both human monocytes and myeloid dendritic cells (Chariot, 2009; Lin et al., 2010). Besides, ‎blocking mTOR in dendritic cells increased IL-12 production and ‎NF-κB activation (Dáňová et al., 2015; Rinkenbaugh and Baldwin, 2016). Our study showed that auraptene attenuates the activity of mTOR and ‎induces NF-κB expression. Thus, it could be suggested that in GBM, the expression of NF-κB, at least in part, is ‎controlled by the mTOR pathway. Conversely, it has been documented that reactive oxygen species (ROS) can exert dual effects by stimulating and inhibiting NF-κB signaling. Our previous study has shown that ROS generation was the primary mechanism of auraptene against U87 cells (Afshari et al., 2019a). Based on this finding, it could be concluded that the upregulation of NF-κB p65 induced by auraptene in the present study seems to be also a consequence of ROS generation in U87 cells.

In summary, the present investigation has yielded initial evidence suggesting that auraptene holds therapeutic potential as a promising natural product in managing tumor metastasis. This conclusion is drawn from the observed inhibitory effects of auraptene on the invasion and migration of GBM cells. The anti-metastatic impacts of auraptene in U87 cells are shown in Figure 9. The portrayal of the detailed mechanisms shows that auraptene inhibits U87 cells, maybe through attenuation of the JNKs and mTOR signaling pathway, diminishing the activities of MMP-2/-9, NF-κB p65 upregulation, and then exerting an anti-metastasis and invasive impact in the U87 cells. Auraptene exhibits potential as a novel natural agent for the therapy of GBM metastasis, owing to its generally non-lethal characteristics and capacity to decrease cell proliferation and limit the metastatic potential of U87 cells.
